# Verapamil Sensitive Ventricular Tachycardia Associated With A Cardiac Hemangioma In The Right Ventricular Outflow Tract

**Published:** 2009-11-01

**Authors:** William J Bonney, Scott R Ceresnak, Shetty Ira, Allan Hordof, Leonardo Liberman

**Affiliations:** The Children's Hospital of New York-Morgan Stanley Children's Hospital-Columbia, New York, NY 10032, USA

**Keywords:** Hemangioma, Ventricular Tachycardia, Verapamil, Triggered Activity, Right Ventricular Outflow Tract (RVOT)

## Abstract

Primary tumors of the heart are rare, but they are often associated with refractory arrhythmias. Vascular tumors of the heart comprise a small minority of primary cardiac tumors. In patients with structurally normal hearts, ventricular tachycardia (VT) originating from the right ventricular outflow tract (RVOT) can be sensitive to adenosine, vagal maneuvers, and calcium channel blockers. In this report, we describe a case of ventricular tachycardia originating from within a hemangioma in the RVOT that was ultimately controlled with verapamil.

## Case report

A 15 year old girl presented with a recurrence of ventricular tachycardia after having been externally cardioverted twice in a 24 hour period at a referring hospital. She had been diagnosed with a cardiac hemangioma when a murmur was noted at 5 years of age on a routine physical exam. Echocardiogram at that time showed a large mass in the RVOT with mild obstruction. Endomyocardial biopsy showed a non-specific vascular proliferation. A histopathologic diagnosis was confirmed when the tumor was resected shortly after the initial presentation. The tumor was infiltrative and encroached on the RVOT free wall and tricuspid valve, and hence was only incompletely resected. She continued to do well, and at 10 years of age, non-sustained polymorphic VT was noted on a routine screening Holter monitor. An EP study was performed and ventricular double extrastimuli (600:290:270) provoked sustained polymorphic VT. At that time, a transvenous single-chamber internal cardioverter defibrillator (ICD) was placed for primary prevention and the patient was started on Nadolol.

**Figure 1:**
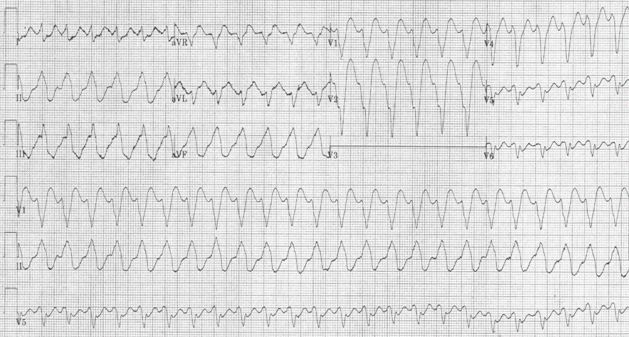
Ventricular Tachycardia (slowed after loading with amiodarone)

She remained asymptomatic for the next five years. Frequent episodes of non-sustained polymorphic VT were noted, but there were no ICD therapies or episodes of sustained VT. At 15 years of age, she presented to the emergency room with palpitations and 12-lead ECG demonstrated wide complex tachycardia at 180 bpm (shown in Figure 1 after amiodarone loading). The ICD was functioning appropriately, but the arrhythmia rate was slower than the defined VT/VF rate of 200 bpm, and hence no therapies were delivered. Tachycardia apparently terminated with amiodarone and she was discharged to home. She presented two days later with the same rhythm. On that occasion, both intravenous amiodarone and lidocaine had no effect on the rhythm, and external cardioversion with 50J restored sinus rhythm.

**Figure 2:**
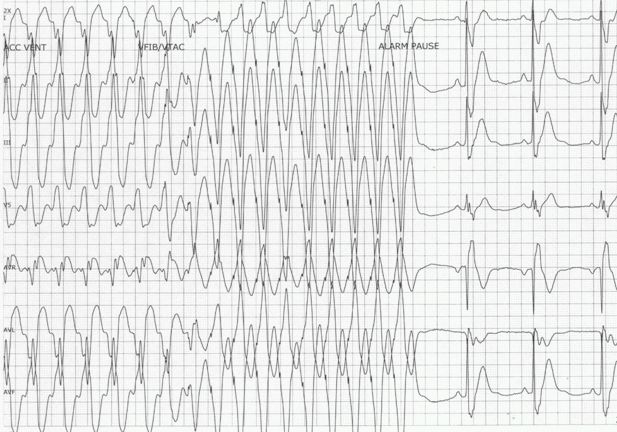
Ventricular tachycardia is terminated with ventricular burst pacing from the ICD

She was then transferred to our institution where Sotalol was initiated at 80 mg twice a day. Ventricular tachycardia recurred, and on both occasions was terminated with antitachycardia pacing using her ICD (Figure 2). Sotalol was discontinued and therapy was transitioned to amiodarone and nadolol. The patient continued to have frequent episodes of VT despite several combinations of antiarrhythmic drugs including amiodarone, lidocaine and esmolol. Tachycardia could be reliably terminated with ventricular burst pacing from the ICD. The patient also reported that on several occasions she was able to successfully terminate tachycardia with vagal maneuvers. Adenosine was given during one episode and did not affect tachycardia.

When daily episodes of VT persisted despite maximal doses of amiodarone and nadolol, antiarrhythmic medications were discontinued for electrophysiology study and possible ablation. Left coronary angiography was performed at the beginning of the case, revealing a coronary fistula from the left coronary artery to the RV with a large contrast-filling vascular mass encircling the left coronary artery. Right coronary angiography demonstrated the same contrast-filling mass encircling the right coronary. The mass was presumed to be residual hemangioma with multiple blood supplies. A trans-esophageal echo with 2D and color doppler was also done, and showed the hemangioma to extend from the mid-portion of the RV septum to just under the pulmonary valve. At EP study, only several beats of nonsustained monomorphic VT were induced in the baseline state. Isoproterenol was infused, and longer runs of sustained monomorphic VT were reliably induced with ventricular burst pacing, ventricular triple extrastimuli, and with rapid atrial pacing. Attempts were made to perform entrainment mapping but were not successful because on some occasions tachycardia terminated with ventricular pacing, and on other attempts reliable capture could not be confirmed. Tachycardia terminated multiple times during the study. Termination was spontaneous and not associated with premature ectopic beats. Tachycardia could reliably be reinitiated on isoproterenol, both with ventricular burst pacing and with ventricular triple extrastimuli (500:320:320:280).

During sustained ventricular tachycardia, 3D mapping with CARTO was initially performed using a Biosense Webster EZ Steer catheter (4mm tip). Earliest areas of ventricular activation in tachycardia were noted along the border of the tumor, and earliest ventricular activation preceeded the QRS by 41 msec. Initial lesions placed along the tumor border were unsuccessful. Pace mapping from these sites showed paced QRS morphology similar to VT morphology in 11 of 12 surface leads. With similar findings at several points on the tumor border, the conclusion was made that tachycardia originated from within the tumor. Attempts to isolate the tumor with a series of lesions using an irrigated Navistar ThermoCool catheter were ultimately unsuccessful and tachycardia persisted. The case was ended and the patient was transferred back to the PICU in sinus rhythm. She underwent an upgrade to a dual chamber ICD with the intention of giving aggressive beta-blocker therapy.

**Figure 3:**
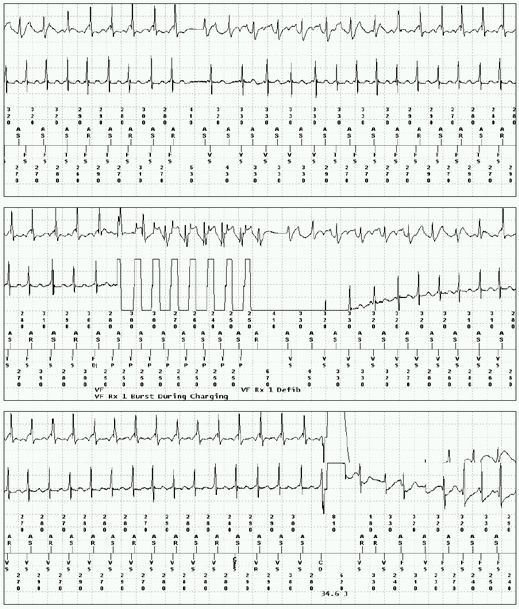
Electrograms stored in the ICD demonstrate sinus tachycardia leading to sustained VT.  Anti-tachycardia pacing during charging is successful in terminating VT briefly before reinitiation and ultimate cardioversion to sinus tachycardia

Sustained tachycardia recurred and intravenous verapamil, which had previously not been tried, was given and tachycardia terminated.  Oral verapamil was continued and there were no further tachycardia recurrences. She was started on oral Verapamil SR 240 mg daily and discharged from the hospital. She continued on Verapamil and did not have any recurrent episodes for one year. Subsequently, during a vigorous exercise the patient experienced two appropriate shocks. She had been noncompliant and was not taking verapamil at the time. Electrograms from the device demonstrated sinus tachycardia prior to an episode of VT (Figure 3).

## Discussion

Cardiac hemangiomas are composed of a benign proliferation of endothelial cells and blood vessels, and they are histologically identical to hemangiomas found elsewhere in the body. Hemangiomas account for less than 3% of primary cardiac tumors [1,2].

Ventricular arrhythmias and sudden cardiac death are associated with a variety of cardiac tumors and are independent of tumor size [3,4]. Tumor associated arrhythmias are often refractory to anti-arrhythmic medical therapies and successful radiofrequency ablation has been described [5].  Tumor resection may be indicated in cases where less invasive therapies have failed [6].

Ventricular tachycardia arising from the RVOT in patients with structurally normal hearts has been characterized by cAMP-mediated triggered activity [7]. These arrhythmias are often sensitive to vagal maneuvers and calcium channel blockers. The arrhythmia described in this case displayed several features of triggered automaticity. Specifically, VT tended to occur while the patient was awake or active. Clinical episodes always initiated with a premature ventricular depolarization and were not pause-dependant. At EP study, isoproterenol was required to produce sustained tachycardia. Tachycardia could also be reliably initiated with ventricular burst pacing, ventricular extrastimuli, and atrial pacing. Finally, the episode provoked by sinus tachycardia during vigorous exercise demonstrated rate-dependant arrhythmia triggering.

## Conclusions

This case illustrates that ventricular tachycardia arising from a hemangioma in the RVOT can act similar to VT arising from the RVOT in patients with structurally normal hearts. Tumor-associated ventricular tachycardias can be difficult to control with medication, and can also be difficult to treat with radiofrequency ablation. Verapamil should be considered before proceeding to tumor resection or other invasive therapies.
